# Superoxide dismutase is associated with cerebral small vessel disease burden and vascular mild cognitive impairment in elderly patients

**DOI:** 10.3389/fnins.2025.1720400

**Published:** 2026-01-05

**Authors:** Xiaohua Xie, Meixi Li, Tianyuan Guan, Zhenjie Teng, Jing Feng, Jing Xu, Qianqian Qi, Yining Xiao, Peiyuan Lv, Yanhong Dong

**Affiliations:** 1 Department of Neurology, Hebei Medical University, Shijiazhuang, China; 2 Department of Neurology, Hebei General Hospital, Shijiazhuang, China; 3 Hebei Key Laboratory of Cerebral Networks and Cognitive Disorders, Hebei General Hospital, Shijiazhuang, China; 4 Department of Rehabilitation, Hebei General Hospital, Shijiazhuang, China; 5 Department of Endocrinology, Hebei General Hospital, Shijiazhuang, China

**Keywords:** cerebral small vessel disease, mild cognitive impairment, oxidative stress, superoxide dismutase, vascular cognitive impairment

## Abstract

**Objective:**

To examine the associations between superoxide dismutase (SOD) levels and the burden of cerebral small vessel disease (CSVD), as well as vascular mild cognitive impairment (VaMCI), in elderly patients.

**Methods:**

In this study, a cohort of 286 elderly individuals was included. Each participant received a comprehensive cognitive assessment. Plasma SOD levels were evaluated. The cumulative CSVD burden was quantified using an ordinal scale ranging from 0 to 4, based on four key imaging indicators of CSVD: white matter hyperintensity, deep cerebral microbleeds, lacunes, and enlarged perivascular spaces in the basal ganglia. To evaluate the associations among SOD levels, CSVD burden, and cognitive function, we employed binary logistic regression analysis, tests for trend, receiver operating characteristic curve analysis, and mediation analysis.

**Results:**

After adjusting for potential confounders, multivariate logistic regression analysis revealed that elevated levels of SOD were significantly associated with a reduced risk of VaMCI (OR: 0.919; 95% CI: 0.896–0.943; *P* < 0.001) and a lower likelihood of severe CSVD burden (OR: 0.980; 95% CI: 0.968–0.992; *P* = 0.001) in elderly patients. Compared with the lowest quartile of SOD levels, the OR for VaMCI in the highest quartile of SOD levels was 0.039 (95% CI: 0.016–0.100; *P* for trend < 0.001) after adjusting for potential confounders. For severe CSVD burden, the corresponding OR was 0.442 (95% CI: 0.214–0.913; *P* for trend = 0.011). Mediation analysis revealed that the severe CSVD burden significantly moderated the relationship between SOD levels and VaMCI.

**Conclusion:**

Elevated SOD levels serve as a protective factor against severe CSVD burden and VaMCI in elderly patients. A portion of the protective effect of increased SOD on VaMCI may be attributed to its role in mitigating the severity of CSVD.

## Introduction

Vascular cognitive impairment (VCI) refers to a broad spectrum of vascular brain abnormalities that lead to any degree of cognitive decline, from mild vascular cognitive impairment (VaMCI) to vascular dementia (VaD) (Mok et al., 2024; Iadecola et al., 2019). VaMCI is widely recognized as an early and potentially reversible stage of VaD (Iadecola et al., 2019; Lissek and Suchan, 2021). Despite the substantial impact of VCI on public health and the economy, the intricate nature of its pathogenesis has hindered the development of sufficiently effective therapeutic interventions (Mok et al., 2024; Rundek et al., 2022).

Preclinical and clinical research findings suggest that cerebral small vessel disease (CSVD) is the primary pathology associated with VCI (Mok et al., 2024; Inoue et al., 2023). Traditionally, structural magnetic resonance imaging (MRI) has been used to visualize tissue damage related to CSVD, which may manifest as lacunes of presumed vascular origin, white matter hyperintensities (WMH), perivascular spaces (PVS), and cerebral microbleeds (CMBs) (Duering et al., 2023; Wardlaw et al., 2013). The total CSVD burden score, derived from a visual rating of these imaging markers, including lacunes, WMH, PVS, and CMBs, exhibits strong construct validity and more accurately reflects the severity of CSVD burden (Duering et al., 2023; Huijts et al., 2013).

Oxidative stress, characterized by an overabundance of reactive oxygen species (ROS) and other reactive molecules, plays a crucial role in the pathological mechanisms underlying VCI (He et al., 2024; Luca and Luca, 2019). Within the context of VCI, oxidative stress can induce endothelial damage, which represents an early event in the progression of this condition (Luca and Luca, 2019). Interestingly, oxidative stress has also been shown to be significantly implicated in the pathogenesis of CSVD (Hannawi, 2024; Grochowski et al., 2018; De Silva and Miller, 2016). In healthy physiological conditions, living organisms sustain a stable redox homeostasis. Endogenous antioxidants within cells, such as superoxide dismutase (SOD), play a critical role in scavenging excess ROS and alleviating oxidative stress-induced damage (Nolfi-Donegan et al., 2020).

SOD serves as an enzymatic antioxidant that catalyzes the conversion of superoxide radicals to hydrogen peroxide within cells, thereby playing a crucial role in antioxidant stress (Islam et al., 2022). SOD deficiency is implicated in the pathogenesis of several neurological disorders, including cognitive impairment and CSVD (Balendra and Singh, 2021; Zhu et al., 2019). In the context of VCI, CSVD and oxidative stress are widely recognized as significant contributors to cognitive decline (Mok et al., 2024; Luca and Luca, 2019). SOD plays a crucial role in mitigating the detrimental effects of ROS, which are generated during events such as inflammation and hypoperfusion injury, both of which are closely associated with CSVD (Hannawi, 2024; Quick et al., 2021). Given the cytoprotective benefits of SOD, significant attention has been directed toward exploring its potential as a biomarker for CSVD and VCI. However, limited research has investigated the association between SOD levels and CSVD burden as well as VaMCI.

Considering the critical role of oxidative stress and microvascular dysfunction in VCI, this study aimed to explore whether SOD is associated with a reduced risk of severe CSVD burden or VaMCI. Additionally, the research examined if the CSVD burden acts as a mediator in the relationship between SOD and VaMCI in elderly patients.

## Materials and methods

### Participants

This retrospective analysis encompassed 286 eligible inpatients consecutively admitted to the Department of Neurology at Hebei General Hospital from August 2019 to April 2022. Inclusion criteria were as follows: (1) age 60 years or older; (2) availability of complete MRI scans, including T1-weighted imaging, T2-weighted imaging, fluid-attenuated inversion recovery, and susceptibility-weighted imaging; and (3) completion of comprehensive cognitive function assessments. Patients were excluded from the study if they had dementia or other conditions that could potentially confound the assessment of cognitive function, such as an acute cerebrovascular event (defined as a clinically diagnosed ischemic stroke or intracerebral hemorrhage occurring within 30 days prior to the assessment), traumatic brain injuries, schizophrenia, hypothyroidism, anxiety disorders, depressive disorders, epileptic disorders, or metabolic encephalopathy.

### Collection of demographic, clinical, and laboratory data

The demographic and clinical data, including age, sex, education level, body mass index (BMI), smoking status, alcohol consumption, blood pressure measurements (both systolic and diastolic), and prior medical history (stroke, diabetes, hypertension, and coronary heart disease), were collected from electronic hospital records or patient interviews. “Current drinking” was defined as the self-reported consumption of any alcoholic beverage at least once per week in the past year.

Laboratory examinations were conducted following an overnight fast of 8 h. The tests included measurements of fasting plasma glucose, total cholesterol (TC), triglyceride, high-density lipoprotein cholesterol (HDL-C), low-density lipoprotein cholesterol (LDL-C), apolipoprotein A1 (ApoA1), apolipoprotein B (ApoB), lipoprotein(a), uric acid, fibrinogen, serum homocysteine, and SOD. Specifically, plasma SOD activity was determined using the pyrogallol autoxidation method with a commercial kit (Autobio Diagnostics, Catalog No. SOD000G). All biochemical analyses were performed on a TBA-FX8 fully automated biochemical analyzer (Canon Medical Systems). The complete specifications of reagent kits are listed in Supplementary material 1.

### Evaluation of CSVD burden

To evaluate the imaging markers of CSVD, a 3.0 Tesla brain MRI (Sigma, GE Healthcare, United States) was utilized. The comprehensive imaging protocol included T1-weighted imaging, T2-weighted imaging, fluid-attenuated inversion recovery, susceptibility-weighted imaging, and diffusion-weighted imaging. The characterization of these imaging markers was conducted in accordance with the Standards for Reporting Vascular Changes on Neuroimaging 2 (STRIVE-2) (Duering et al., 2023). A total CSVD score was determined on an ordinal scale from 0 to 4, incorporating four key imaging markers: lacunes, WMH, PVS, and CMBs (Duering et al., 2023; Huijts et al., 2013). The Fazekas rating scale was employed to assess the severity of periventricular and deep WMH (Fazekas et al., 1993). The Microbleed Anatomical Rating Scale was utilized to evaluate CMBs (Gregoire et al., 2009). The number of PVS or lacunes was documented. A score of one point was assigned for each of the following criteria: a Fazekas score of 3 for periventricular WMH or ≥ 2 for deep WMH, the presence of at least one lacune, one or more deep CMBs, and a count of ≥ 11 PVS in the basal ganglia. The total CSVD score was used to evaluate the overall burden of CSVD (Huijts et al., 2013; Staals et al., 2015). Total CSVD scores exceeding 2 were classified as indicative of severe CSVD burden (Kim et al., 2021; Teng et al., 2022).

### Assessment of vascular mild cognitive impairment

In this study, all patients underwent a comprehensive series of neuropsychological assessments. These evaluations included the standardized Chinese (Beijing) version of the Montreal Cognitive Assessment (MoCA), assessments of both basic and instrumental activities of daily living (ADL and IADL), the 14-item Hamilton Anxiety Rating Scale (HAMA), and the 24-item Hamilton Depression Rating Scale (HAMD). Cognitive impairment was objectively confirmed based on the following criteria from neuropsychological tests: MoCA scores ( ≤ 13 for individuals without formal education, ≤ 19 for those with 1–6 years of schooling, and ≤ 24 for those with 7 or more years of education), HAMA score < 6, and HAMD score < 8 (Lu et al., 2011). The diagnosis of VaMCI was established according to previously published criteria. These criteria included: (a) Objective evidence of cognitive decline; (b) clinical features consistent with vascular etiology; (c) sufficient cerebrovascular disease to account for the cognitive deficits; and (d) absence of dementia, as indicated by normal performance in basic ADL and an IADL score below 10 (Skrobot et al., 2018; van der Flier et al., 2018).

### Statistical analysis

Continuous variables were summarized using mean and standard deviation or median and interquartile range, depending on the distribution of data. Categorical variables were presented as proportions or percentages. For continuous variables, comparisons were carried out using Mann-Whitney U tests or independent samples *t*-tests based on the normality of data distribution. Categorical variables were analyzed using χ^2^ tests. Variables with a *P*-value < 0.1 in the initial univariate analyses were incorporated into multivariate binary logistic regression models to identify independent risk factors for VaMCI or severe CSVD burden. To evaluate trends, SOD levels were categorized into quartiles. The receiver operating characteristic (ROC) curve for SOD levels was constructed, and the optimal cut-off point was determined based on the maximum Youden Index. Statistical significance was set at *P* < 0.05. All statistical analyses were performed using SPSS version 26.0 (IBM Corporation, Armonk, NY).

For mediation analysis, R version 4.1.0 (R Foundation for Statistical Computing, Vienna, Austria) was utilized with the bruceR and mediation packages. This analysis aimed to assess whether severe CSVD burden mediated the relationship between SOD levels and VaMCI. The software automatically selected the appropriate model type and performed mean centering before building the model. Each analysis utilized 5,000 bootstrap samples to ensure robust effect estimation.

## Results

### Participants characteristics

This retrospective study included 286 eligible participants, consisting of 126 individuals diagnosed with VaMCI and 160 with normal cognitive function. The median age of the participants was 69 years, with 54.5% being male. The prevalence of VaMCI among elderly patients was 44.1%. Table 1 summarizes the characteristics of patients in both groups. Compared to those with normal cognitive function, participants in the VaMCI group were significantly older and had lower educational levels (*P* < 0.05). Additionally, the VaMCI group exhibited a higher frequency of severe CSVD burden compared to the normal cognitive group (*P* < 0.05). Regarding biochemical markers, individuals in the VaMCI group showed elevated levels of fibrinogen and serum homocysteine, while they had reduced levels of HDL-C, ApoA1, and SOD (*P* < 0.05).

**TABLE 1:** Characteristics of participants between normal cognitive group and VaMCI group.

Variable	Total (*n* = 286)	Normal cognitive group (*n* = 160)	VaMCI group (*n* = 126)	*P-*value	Effect size (95% CI)
Age, median (IQR), year	69 (65–74)	68 (64–73)	71 (67–75)	< 0.001*	ΔM: 3.00 (1.00, 4.00)
Sex (male), n (%)	156 (54.5)	81 (50.6)	75 (59.5)	0.133	φ: 0.09 (–0.03, 0.20)
Education, median (IQR), year	9 (6–12)	9 (6–12)	9 (6–11)	0.018*	ΔM: 0.00 (0.00, 3.00)
BMI, median (IQR), kg/m^2^	25.3 (23.2–27.3)	25.4 (23.2–27.3)	25.2 (23.2–27.8)	0.933	ΔM: 0.02 (–0.77, 0.82)
Current smoking, n (%)	53 (18.5)	27 (16.9)	26 (20.6)	0.417	ΔM: 2.00 (–3.00, 7.00)
Current drinking, n (%)	41 (14.3)	25 (15.6)	16 (12.7)	0.483	ΔM: 0.00 (–3.00, 2.00)
Hypertension, n (%)	196 (68.5)	111 (69.4)	85 (67.5)	0.729	φ: 0.05 (–0.07, 0.16)
SBP, median (IQR), mmHg	140 (127–152)	138 (126–152)	143 (127–152)	0.408	φ: 0.04 (–0.08, 0.16)
DBP, median (IQR), mmHg	81 (74–88)	81 (74–88)	80 (75–87)	0.785	φ: 0.02 (–0.10, 0.14)
Diabetes, n (%)	84 (29.4)	42 (26.3)	42 (33.3)	0.192	φ: 0.08 (–0.04, 0.19)
Coronary heart disease, n (%)	59 (20.6)	34 (21.3)	25 (19.8)	0.770	φ: 0.02 (–0.10, 0.13)
History of stroke, n (%)	115 (40.2)	58 (36.3)	57 (45.2)	0.124	φ: 0.09 (–0.03, 0.21)
Fasting plasma glucose, median (IQR), mmol/L	5.08 (4.60–6.00)	5.08 (4.59–5.86)	5.08 (4.60–6.73)	0.493	ΔM: 0.08 (–0.16, 0.34)
TC, median (IQR), mmol/L	4.31 (3.61–5.04)	4.36 (3.70–5.11)	4.12 (3.54–4.91)	0.107	ΔM: –0.19 (–0.42, 0.04)
Triglyceride, median (IQR), mmol/L	1.14 (0.86–1.57)	1.18 (0.88–1.60)	1.07 (0.80–1.54)	0.127	ΔM: –0.10 (–0.22, 0.03)
HDL-C, median (IQR), mmol/L	1.10 (0.94–1.31)	1.14 (0.98–1.37)	1.03 (0.89–1.25)	0.001*	ΔM: –0.11 (–0.17, –0.05)
LDL-C, median (IQR), mmol/L	2.73 (2.21–3.32)	2.78 (2.25–3.35)	2.69 (2.17–3.31)	0.391	ΔM: –0.07 (–0.25, 0.10)
ApoA1, median (IQR), g/L	1.27 (1.10–1.41)	1.29 (1.17–1.47)	1.20 (1.02–1.36)	< 0.001*	ΔM: –0.12 (–0.18, –0.06)
ApoB, median (IQR), g/L	0.73 (0.63–0.88)	0.71 (0.63–0.89)	0.76 (0.63–0.88)	0.652	ΔM: 0.01 (–0.03, 0.06)
Lipoprotein (a), median (IQR), mg/L	191.8 (101.8–332.7)	182.9 (88.5–313.0)	204.1 (108.4–356.6)	0.066	ΔM: 29.40 (–2.20, 65.60)
Uric acid, median (IQR), umol/L	285.5 (240.3–338.1)	288.3 (242.3–341.6)	280.8 (235.5–331.7)	0.450	ΔM: –7.20 (–24.80, 11.10)
Fibrinogen, median (IQR), g/L	2.80 (2.41–3.31)	2.75 (2.34–3.19)	2.86 (2.55–3.55)	0.020*	ΔM: 0.18 (0.03, 0.34)
Serum homocysteine, median (IQR), umol/L	13.2 (10.6–16.5)	12.6 (9.6–15.4)	14.1 (11.4–19.0)	0.003*	ΔM: 1.70 (0.60, 2.80)
SOD, median (IQR), U/mL	141.7 (127.5–154.0)	148.1 (140.3–158.9)	129.1 (119.7–138.6)	< 0.001*	ΔM: –20.10 (–23.70, –16.30)
Severe CSVD burden, n (%)	130 (45.5)	36 (22.5)	94 (74.6)	< 0.001*	φ:0.52 (0.42, 0.62)

*Denotes significance at a *P-*value of < 0.05. Effect sizes: ΔM = Hodges-Lehmann median difference (VaMCI group minus normal group) for continuous variables; φ = Phi coefficient for categorical variables. Values in parentheses represent 95% confidence intervals. VaMCI, vascular mild cognitive impairment; BMI, body mass index; SBP, systolic blood pressure; DBP, diastolic blood pressure; TC, total cholesterol; HDL-C, high density lipoprotein cholesterol; LDL-C, low density lipoprotein cholesterol; ApoA1, apolipoprotein A1; ApoB, apolipoprotein B; SOD, superoxide dismutase; CSVD, cerebral small vessel disease.

### Superoxide dismutase levels and vascular mild cognitive impairment

In unadjusted logistic regression analysis, elevated SOD levels were associated with a decreased likelihood of VaMCI (OR: 0.926; 95% CI: 0.908–0.945; *P* < 0.001). This association remained statistically significant in multivariate logistic regression analysis (OR: 0.919; 95% CI: 0.896–0.943; *P* < 0.001), after adjusting for potential confounders (Table 2). SOD levels exhibited a negative relationship with the risk of VaMCI. Compared to the lowest quartile of SOD levels, individuals in the highest quartile demonstrated a significantly reduced risk of VaMCI (OR: 0.039; 95% CI: 0.016–0.100; *P* for trend < 0.001) after controlling for age, education, hypertension, and serum homocysteine (Table 3).

**TABLE 2:** The logistic regression analyses between possible predictors and VaMCI in elderly patients.

	Univariable analysis		Multivariable analysis	
Variable	OR (95% CI)	*P-*value	OR (95% CI)	*P-*value
Age	1.093 (1.048–1.141)	<0.001	1.077 (1.013–1.146)	0.018
Education	0.944 (0.893–0.998)	0.042	0.921 (0.849–0.999)	0.046
HDL-C	0.223 (0.088–0.561)	0.001	0.962 (0.139–6.639)	0.969
ApoA1	0.112 (0.040–0.318)	<0.001	0.132 (0.015–1.159)	0.068
Lipoprotein (a)	1.001 (1.000–1.002)	0.068	1.001 (0.999–1.003)	0.286
Fibrinogen	1.349 (1.020–1.784)	0.036	1.032 (0.706–1.508)	0.872
Serum homocysteine	1.038 (1.008–1.070)	0.014	1.019 (0.978–1.062)	0.361
SOD	0.926 (0.908–0.945)	<0.001	0.919 (0.896–0.943)	<0.001
Severe CSVD burden	10.12 (5.86–17.48)	<0.001	13.48 (6.34–28.68)	<0.001

VaMCI, vascular mild cognitive impairment; HDL-C, high density lipoprotein cholesterol; LDL-C, low density lipoprotein cholesterol; ApoA1, apolipoprotein A1; SOD, superoxide dismutase; CSVD, cerebral small vessel disease.

**TABLE 3:** ORs (and 95% CIs) of VaMCI and severe CSVD burden according to quartiles of SOD levels.

	SOD levels, median (range)				
Model	Quartile 1 119.8 (≤ 127.5)	Quartile 2 134.5 (127.6–141.6)	Quartile 3 146.3 (141.7–154.0)	Quartile 4 164.4 (≥ 154.1)	*P-*value for trend
**VaMCI**					
Model 1	1.00 (reference)	0.244 (0.112–0.530)	0.053 (0.023–0.122)	0.041 (0.017–0.098)	<0.001
Model 2	1.00 (reference)	0.241 (0.108–0.536)	0.053 (0.022–0.127)	0.042 (0.017–0.104)	<0.001
Model 3	1.00 (reference)	0.262 (0.117–0.588)	0.058 (0.024–0.139)	0.039 (0.016–0.100)	<0.001
Effect size (Q4 vs. Q1)					*h* = 1.453 (1.125–1.781)
**Severe CSVD burden**					
Model 1	1.00 (reference)	0.823 (0.426–1.591)	0.454 (0.233–0.884)	0.411 (0.209–0.807)	0.003
Model 2	1.00 (reference)	0.871 (0.445–1.707)	0.505 (0.255–0.998)	0.486 (0.243–0.971)	0.018
Model 3	1.00 (reference)	0.954 (0.476–1.914)	0.547 (0.027–1.106)	0.442 (0.214–0.913)	0.011
Effect size (Q4 vs. Q1)					*h* = 0.440 (0.112–0.767)

Model 1: Unadjusted; Model 2: Adjusted for age and education. Model 3: Adjusted for age, education, hypertension, and serum homocysteine. Cohen’s h effect sizes are reported for the comparison between the highest (Q4) and lowest (Q1) SOD quartiles, with 95% confidence intervals in parentheses.

### Superoxide dismutase levels and severe CSVD burden

In unadjusted logistic regression analysis, elevated SOD levels were associated with a decreased likelihood of severe CSVD burden (OR: 0.980; 95% CI: 0.968–0.992; *P* = 0.001). This association remained statistically significant in multivariate logistic regression analysis (OR: 0.979; 95% CI:0.965–0.993; *P* = 0.003), after adjusting for potential confounders (Table 4). SOD levels exhibited a negative relationship with the risk of severe CSVD burden. Compared to the lowest quartile of SOD levels, individuals in the highest quartile demonstrated a significantly reduced risk of severe CSVD burden (OR: 0.442; 95% CI: 0.214–0.913; *P* for trend = 0.011) after controlling for age, education, hypertension, and serum homocysteine (Table 3).

**TABLE 4:** The logistic regression analyses between possible predictors and severe CSVD burden in elderly patients.

Variable	Univariable analysis		Multivariable analysis	
	OR (95% CI)	*P-*value	OR (95% CI)	*P-*value
Age	1.078 (1.034–1.124)	<0.001	1.083 (1.031–1.137)	0.001
Sex (male)	2.008 (1.247–3.233)	0.004	1.545 (0.852–2.800)	0.152
Education	0.981 (0.929–1.036)	0.494	–	–
BMI	1.015 (0.949–1.085)	0.667	–	–
Current smoking	1.735 (0.950–3.167)	0.073	1.700 (0.805–3.588)	0.164
Current drinking	0.929 (0.477–1.809)	0.829	–	–
Hypertension	1.692 (1.014–2.824)	0.044	2.036 (1.099–3.775)	0.024
SBP	1.012 (1.000–1.024)	0.055	1.010 (0.996–1.024)	0.146
DBP	1.012 (0.993–1.032)	0.218	–	–
Diabetes	1.701 (1.018–2.840)	0.042	1.332 (0.653–2.717)	0.430
Coronary heart disease	0.932 (0.524–1.658)	0.810	–	–
History of stroke	1.670 (1.036–2.690)	0.035	1.393 (0.815–2.383)	0.226
Fasting plasma glucose	1.139 (1.003–1.293)	0.044	1.143 (0.957–1.364)	0.140
TC	0.865 (0.682–1.098)	0.235	–	–
Triglyceride	1.042 (0.839–1.294)	0.712	–	–
HDL-C	0.323 (0.132–0.789)	0.013	0.309 (0.066–1.443)	0.135
LDL-C	0.892 (0.651–1.223)	0.478	–	–
ApoA1	0.389 (0.151–1.001)	0.050	1.727 (0.328–9.101)	0.519
ApoB	1.063 (0.311–3.633)	0.922	–	–
Lipoprotein (a)	1.000 (0.999–1.001)	0.822	–	–
Uric acid	1.002 (0.999–1.004)	0.254	–	–
Fibrinogen	1.144 (0.881–1.485)	0.313	–	–
Serum homocysteine	1.051 (1.017–1.086)	0.003	1.046 (1.010–1.084)	0.012
SOD	0.980 (0.968–0.992)	0.001	0.979 (0.965–0.993)	0.003

CSVD, cerebral small vessel disease; BMI, body mass index; SBP, systolic blood pressure; DBP, diastolic blood pressure; TC, total cholesterol; HDL-C, high density lipoprotein cholesterol; LDL-C, low density lipoprotein cholesterol; ApoA1, apolipoprotein A1; ApoB, apolipoprotein B; SOD, superoxide dismutase.

### Mediation analysis

**FIGURE 1:**
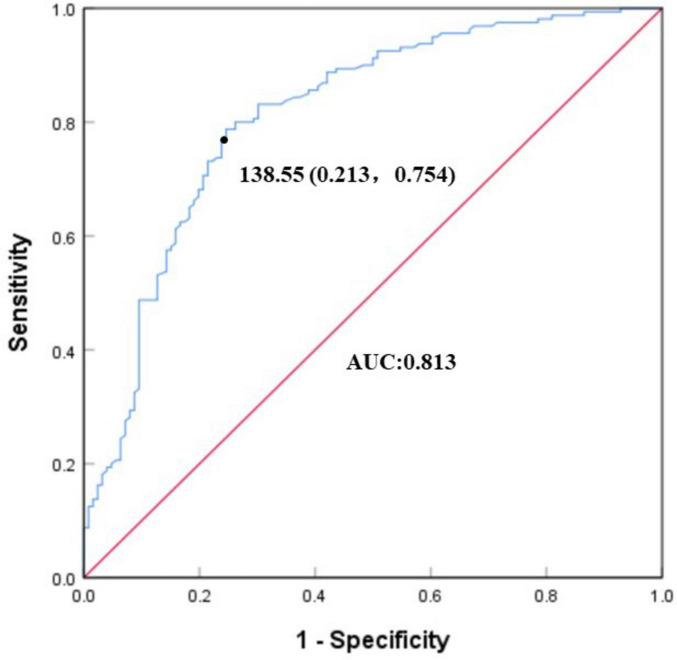
ROC curve of SOD levels for VaMCI. The optimal threshold for SOD levels in participants with VaMCI was determined to be 138.55. The specificity was 0.787 (1–0.213) and sensitivity was 0.754. The area under the curve (AUC) was 0.813.

**FIGURE 2:**
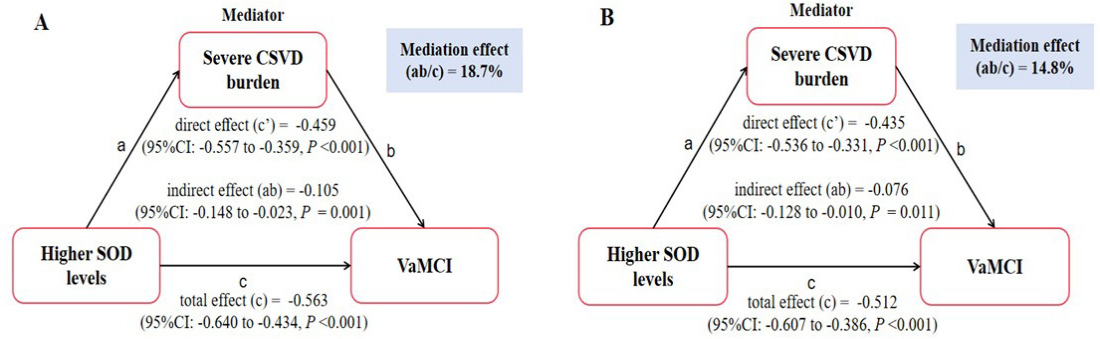
Mediation analysis is shown for the presence of severe CSVD burden as a mediator in the relation between SOD levels and VaMCI. **(A)** Unadjusted. **(B)** Adjusted for age, education, hypertension, and serum homocysteine.

The optimal threshold for SOD levels in participants with VaMCI was determined to be 138.55, with an area under the curve (AUC) of 0.813 (Figure 1). Based on this threshold, participants were categorized into two groups: those with elevated SOD levels and those with lower SOD levels. Mediation analysis revealed a significant direct effect (c’ = –0.459, *P* < 0.001) and total effect (c = –0.563, *P* < 0.001) between elevated SOD levels and VaMCI. When severe CSVD burden was introduced as a mediator, the indirect effect (ab = –0.105, *P* = 0.001) became significant, accounting for 18.7% of the total effect (Figure 2A). After adjusting for potential confounders, the indirect effect of severe CSVD burden remained significant, contributing 14.8% to the total effect (Figure 2B).

## Discussion

In this study, we conducted a retrospective analysis to investigate the relationship between SOD levels and both VaMCI and CSVD burden in elderly patients. Our findings indicate that elevated SOD levels serve as a protective factor against severe CSVD burden and VaMCI in elderly patients, even after adjusting for potential confounding variables. These negative associations were more pronounced in patients with higher SOD levels. Additionally, our results suggest that the effect of increased SOD levels on VaMCI is partly mediated by the presence of a severe CSVD burden, potentially indicating that higher SOD levels may mitigate severe CSVD burden, thereby possibly reducing the risk of VaMCI. This underscores the critical role of SOD in modulating the progression of these conditions.

Oxidative stress has been implicated in the pathogenesis of various neurological disorders, including VCI (Luca and Luca, 2019) and CSVD (Grochowski et al., 2018). In elderly populations, the progressive accumulation of oxidative damage may exacerbate the severity of these conditions, potentially contributing to cognitive decline and other neurological manifestations. SOD is a vital antioxidant enzyme that serves as a critical indicator of the capacity to scavenge oxygen free radicals, thereby playing an indispensable role in mitigating oxidative stress (Fukai and Ushio-Fukai, 2011; Fanoudi et al., 2019).

Oxidative stress, intricately associated with inflammation, has been identified as a critical determinant in the pathogenesis and progression of CSVD (De Silva and Miller, 2016). The accumulation of localized ROS, which originates during the early stages of brain vascular injury, can both precipitate and result from mitochondrial dysfunction, endothelial dysfunction, inflammatory responses, and cellular apoptosis (Grochowski et al., 2018). Oxidative stress progressively intensifies with advancing age and in the presence of multiple CSVD risk factors (Liguori et al., 2018), further underscoring its pivotal role in the pathology of CSVD. Several studies demonstrated a significant association between SOD and CSVD. Zhu et al. found that decreased SOD levels act as an independent risk factor for WMH (Zhu et al., 2019). Furthermore, research has indicated that specific antioxidants may alleviate CSVD or CSVD-related cognitive impairments through the enhancement of SOD levels (Jiang et al., 2021; Wang and Hu, 2018). However, to the best of our knowledge, no prior studies have examined the relationship between SOD levels and the CSVD burden, which serves as a comprehensive indicator of CSVD severity (Duering et al., 2023). In our study, we observed that elevated SOD levels serve as a protective factor against severe CSVD burden in elderly patients. The negative correlation was more pronounced in patients with higher SOD levels, suggesting a potential dose-response relationship.

Interestingly, oxidative stress also has been demonstrated to be critically involved in the pathogenesis of VCI (Rundek et al., 2022; Luca and Luca, 2019). An increasing body of evidence suggests that low levels of SOD are closely associated with the onset and progression of cognitive impairment (Zhu et al., 2019; Fracassi et al., 2021). The underlying mechanisms are multifaceted and complex. Previous studies have shown that SOD deficiency accelerates amyloid β oligomerization or promotes tau protein phosphorylation in Alzheimer’s disease (AD) model mice (Murakami et al., 2011; Massaad et al., 2009; McLimans et al., 2019), suggesting a critical pathway by which SOD deficiency may influence VCI. More importantly, elevated levels of SOD may alleviate oxidative stress, suppress inflammatory responses, and safeguard the integrity of the blood-brain barrier (Fukai and Ushio-Fukai, 2011; Zhong et al., 2008; Ishihara et al., 2015), which are essential for reducing the risk of VCI. A study demonstrated that low SOD levels were associated with an increased risk of cognitive impairment after mild acute ischemic stroke, an important subtype of VCI (Zhang et al., 2022). Conversely, a community-based prospective study reported that higher SOD activity was linked to an elevated risk of cognitive decline in older Chinese adults (Sun et al., 2019). However, this study did not further differentiate between subtypes and degrees of cognitive impairment.

Importantly, previous studies investigating the relationship between SOD and cognitive impairment have not adequately considered the impact of small cerebral vascular disease, which exerts a significant influence on cognitive impairment. This omission may have resulted in an incomplete understanding of the underlying mechanisms contributing to cognitive impairment. Our study identified low SOD levels as an independent risk factor for VaMCI. Further analysis demonstrated that, after adjusting for potential confounding factors, part of the association between SOD levels and VaMCI was mediated by the presence of a severe CSVD burden.

The primary strength of our study lies in its consideration of CSVD when examining the relationship between SOD levels and VaMCI in elderly patients. However, several limitations must be acknowledged. Firstly, the retrospective nature of this study design precludes a definitive establishment of causality. While the research identified an association between SOD levels and both CSVD burden and VaMCI in elderly patients, the direction of causation remains unclear. It is plausible that changes in SOD levels are a consequence of CSVD and VaMCI progression rather than an initiating factor. Secondly, the retrospective design introduces potential selection bias. The patient cohort was likely drawn from specific hospitals or medical records, which may not be representative of the broader elderly population affected by CSVD and VaMCI. Thirdly, unaccounted confounding factors present a significant challenge. Numerous variables could influence the relationship between SOD, CSVD, and VaMCI, and accurately measuring and controlling for all these confounders in a retrospective study can be difficult. Future prospective studies are necessary to address these limitations.

## Conclusion

In conclusion, our study provides evidence that elevated SOD levels are inversely associated with the risk of VaMCI or CSVD burden in elderly patients. Notably, severe CSVD burden significantly moderates the relationship between higher SOD levels and VaMCI. These findings suggest that a portion of the protective effect of elevated SOD levels on VaMCI may be attenuated by the exacerbation of CSVD burden. Elevated SOD levels represent a promising therapeutic target for protecting elderly patients from VaMCI and CSVD. Further research is warranted to explore potential strategies aimed at enhancing SOD activity, which could ultimately contribute to improved neurological health and cognitive function in older adults.

## Data Availability

The raw data supporting the conclusions of this article will be made available by the authors, without undue reservation.
