# Supplementary material for: Superoxide dismutase is associated with cerebral small vessel disease burden and vascular mild cognitive impairment in elderly patients
Source: Front Neurosci. 2026 Jan 5;19:1720400. doi: 10.3389/fnins.2025.1720400 (PMC12812885; doi:10.3389/fnins.2025.1720400)
Supplement: Supplementary file 1 [file Data_Sheet_1.doc]

**Supplementary Material 1: Detailed Laboratory Methods and Reagent Specifications**

1. Instrumentation

All biochemical measurements were performed on a TBA-FX8 fully automated biochemical analyzer from Canon Medical Systems (Dalian) Co., Ltd., China.

2. Reagent Kits and Assay Principles

All commercial reagent kits were supplied by Autobio Diagnostics Co., Ltd. (Zhengzhou, China). The specific details for each biomarker are as follows:

The activity of superoxide dismutase (SOD) in plasma was determined using the pyrogallol autoxidation method (Catalog No. SOD000G).

Serum homocysteine was measured via an enzymatic cycling assay (Catalog No. HCY100G).

The lipid profile was assessed using enzymatic colorimetric methods: total cholesterol (TC) by the CHOD-PAP method (Catalog No. CHO000G), triglycerides (TG) by the GPO-PAP method (Catalog No. TG0000G). High-density lipoprotein cholesterol (HDL-C) and low-density lipoprotein cholesterol (LDL-C) were measured by direct methods (Catalog No. HDL100G and LDL000G, respectively).

Apolipoprotein A1 (ApoA1) and apolipoprotein B (ApoB) were quantified using immunoturbidimetric assays (Catalog No. AA1000G and APB000G, respectively).

Uric acid (UA) was assayed using the uricase method (Catalog No. UA000G).

3. Sample Processing and Quality Control

All procedures were conducted following the manufacturer‘s protocols under strict internal quality control standards in the Central Laboratory of Hebei General Hospital.
